# Esophageal extraskeletal neoplasm Ewing's sarcoma: Case report

**DOI:** 10.1016/j.ijscr.2022.107399

**Published:** 2022-07-09

**Authors:** Hina Khalid, Niaz Hussain, Rafay Shamshad

**Affiliations:** a Dow University of Health Sciences, Karachi, Pakistan; b Department of Thoracic Surgery, Ojha Institute of Chest Disease, Pakistan; c Department of Thoracic Surgery, Pakistan

**Keywords:** Extraosseous Ewing sarcoma (EES)

## Abstract

**Introduction and importance:**

Ewing sarcomas are a group of small round cell tumors that occur predominantly in the long bones as well as in extraosseous locations such as the extremities, trunk, and retroperitoneum (Gier, 1997) [2]. Extraosseous Ewing sarcoma (EES) is a type of small round cell tumor that occurs in soft tissues. I rare cases, EES occurs in the esophagus (Maesawa et al., 2002; Johnson et al., 2010) [1,3]. Ewing's sarcoma is a rare and highly aggressive cancer most frequently arising in people under 20 years of age. We report an uncommon case of primary paraesophageal Ewing's sarcoma in a 25-year-old female.

**Case presentation:**

A 26 years old Asian female referred primarily for surgical treatment due to esophageal cancer detected on her diagnostic investigations and revealed a primary tumor located near the gastroesophageal junction. Based on the results of diagnostic investigations which confirmed the possibility of the tumor Ewing sarcoma of esophagus, which was biopsy and immune histochemical stain proven the patient was qualified for surgical treatment. She underwent Mckewon esophagectomy on October 2021 for Ewing sarcoma of esophagus. She was first followed with neoadjuvant intravenous chemotherapy, after taking three cycles of neoadjuvant chemo showed good response in CT scan the patient underwent Mckewon esophagectomy, post op recovery was smooth she underwent 2 cycles of adjuvant chemotherapy after four months of surgery. Her followup visit was uneventful.

**Clinical discussion:**

Ewing's sarcoma is the second most frequent primary malignant bone cancer, after osteosarcoma. It was first described by James Ewing in 1921, as an undifferentiated tumor developing in the diaphysis of the ulna of a young female patient (Ushigome et al., 2002) [6]. Ewing sarcoma/primitive neuroectodermal tumor (ES/PNET), previously thought to be separate tumors, is now treated as the same tumor; both have similar immunohistochemical characteristics and chromosomal translocation (Maesawa et al., 2002) [1]. They are malignant tumors composed of undifferentiated small round cells, usually affecting children, adolescents, and young adults (Kondo et al., 2005) [7]. Generally ES/PNET affects the bones and deep soft tissues (Soulard et al., 2005) [8], although other organs such as the pancreas, small bowel, esophagus, kidneys, prostate, ovaries, vagina and rectovaginal septum have been reported; this is termed as extraskeletal ES/PNET (Bloom et al., 1995) [9]. To the best of our knowledge, only 5 cases of gastric ES/PNET have been reported in the English language literature.

Extraskeletal Ewing's sarcoma is a very rare disease, accounting for 6 %–47 % of all cases of Ewing's sarcoma. It is mainly diagnosed in the trunk, extremities, retroperitoneum, and head and neck region. Patients with extraosseous Ewing's sarcoma are more likely to be older, female, and not of Caucasian origin. An extraskeletal origin of the disease is correlated to poor prognosis (Siegel et al., 1988; Granowetter and West, 1997; Ushigome et al., 2002) [[4], [5], [6]]. We present an uncommon case of extraskeletal Ewing's sarcoma, and discuss its rare presentation and evolution. To our knowledge, this is the first reported case of paraesophageal primary Ewing's sarcoma and primitive neuroectodermal tumor.

Adenocarcinoma and squamous cell carcinoma account for the vast majority of esophageal malignancies. Other malignancies known to occur in the esophagus include melanoma, sarcoma, and lymphoma. Among the sarcomas, carcinosarcoma is the commonest with both carcinomatous and sarcomatous elements followed by leiomyosarcoma of mesenchymal origin. Other sarcomas reported in the literature are liposarcoma, synovial sarcoma, myxofibrosarcoma, Ewing's sarcoma, granulocytic sarcoma, histiocytic sarcoma, schwannoma rhabdomyosarcoma, and epithelioid sarcoma.

**Conclusion:**

Ewing sarcoma is a rare entity among all esophageal malignancies. It presents as an exophytic mass, and in this case, it has presented as a mass occluding the lumen of esophagus. Most of these tumors present in locally advanced and disseminated condition, one of the reasons being difficulty and hence delay in diagnosis. In spite of best efforts, a group among them remains to be histologically uncharacterized.

## Highlights

• Extraskeletal Ewing's sarcoma is a very rare disease, accounting for 6 %–47 % of all cases of Ewing's sarcoma.
• Ewing sarcoma is a rare entity presenting as an exophytic mass.
• Ewing's sarcoma of the paraesophagus is a very unusual condition.
• Ewing's sarcoma is the second most frequent primary malignant bone cancer, after osteosarcoma.
• Ewing sarcoma an extraskeletal origin of the disease

## 1 Discussion

Ewing's sarcoma of the paraesophagus is a very unusual condition. Only three cases of esophageal Ewing's sarcoma have been published in the literature to yet, to our knowledge. The majority of occurrences (10–11 %) occurred in adults over the age of 20. Monomorphic round cells with small hyperchromatic nuclei, inconspicuous nucleoli, sparse cytoplasm, and large necrotic regions define both skeletal and extraskeletal Ewing's sarcomas. It's difficult to make a diagnosis for such a rare occurrence. Ewing's sarcoma shares histological and immunophenotypic characteristics with other juvenile small round cell cancers. As a result, an enlarged panel of immunohistochemical tests, fluorescence in situ hybridization, and reverse transcription polymerase chain reaction (RT-PCR) are required to rule out additional diseases such as neuroblastoma, lymphoblastic lymphoma, poorly differentiated synovial sarcoma, and so on [12].

The EWSR1 gene is one of the genes most sensitive to translocation in soft tissue tumors and encodes the EWS protein, which is a member of a growing family of highly conserved RNA-binding proteins mediating interaction with RNA or single-stranded DNA. The codified protein takes part in transcriptional regulation for specific genes and in mRNA splicing. Specifically, EWRS1 is involved in transcription initiation. Concerning EWSR1 breakpoints, the main areas susceptible to breakage are EWSR1 exons 7, 8, 9, or 10 [15].

Surgery, wherever possible, remains to be the mainstay of treatment [13]. Esophagectomy/esophagogastrectomy is the surgery of choice. Even if metastases are present, a palliative resection can still be performed [13]. Endoscopic resection is another surgical option available [14]. The role of adjuvant radiotherapy and chemotherapy is controversial [13]. Palliative procedures like stenting to relieve dysphagia improve quality of life [16,17].

### 1.1 The present case in the context of the literature

Sarcoma is a rare entity among all esophageal malignancies. It presents as an exophytic mass, and in this case, it has presented as a stricture esophagus. Most of these tumors present in locally advanced and disseminated condition, one of the reasons being difficulty and hence delay in diagnosis. In spite of best efforts, a group among them remains to be histologically uncharacterized. Here, we report a case of malignant spindle cell tumor of esophagus, a cause for a stricture esophagus. A definitive histopathological diagnosis could not be achieved.

Even in the case of inoperable disease, palliative resection has a role to play in terms of treatment. The importance of strong local treatment should be highlighted in light of locoregional failure. Endoscopically, polypoid and exophytic masses [18], as well as ulcerating tumors [19], are present. Large intramural masses with ulceration/tracking, expansile intraluminal masses, or areas of luminal constriction may be seen on barium scans [20]. Stricture esophageal stricture is a rare complication. CT/MRI imaging may reveal an intramural mass that is not enhancing uniformly [23]. Submucosal esophageal tumors, which would ordinarily require open biopsy for diagnosis, are one of the criteria for endoscopic ultrasound and its guided biopsy or fine needle aspiration cytology. As a result, the period between diagnosis and treatment may be reduced [21].

## 2 Case presentation

A 26 years old Asian female referred primarily for surgical treatment due to esophageal cancer detected on her diagnostic investigations and revealed a primary tumor located near the gastroesophageal junction. She was on clinical examination was anemic and her abdominal examination was unremarkable so she was investigated for her abdominal pain and her CT scan abdomen was done that revealed a heterogeneously enhancing mass lesion seen arising from the lower end of esophagus projecting into the lumen.it measures about 6.3 ∗ 8 cm in AP and transverse dimension, lymph node also noted in lesser sac, appearance are suggestive of lesion involving the GE junction and proximal stomach no pulmonary and hepatic metastases noted.

The PET–CT was performed revealing no bony metastases at the time of scan. She underwent for upper GI endoscopy that showed mass partially obstructing the lumen of esophagus biopsy were taken.

Biopsy report came out to be positive for immunohistochemical stains CD99, Cyclin D-1 and NKX 2.2, Ewing sarcoma is a possibility.

Other immunohistochemical stains were performed which showed synaptophysin positive, CD99 positive, NKX2 positive, Cyclin D-1 positive, and cytokeratin CAM 5.2 positive, and interpretation translocation of 23Q-12 is not detected. Based on the results of diagnostic investigations which confirmed the possibility of the tumor Ewing sarcoma of esophagus, she was first followed with neoadjuvant intravenous chemotherapy, after taking three cycles of neoadjuvant chemo showed good response in CT scan and endoscopy the patient underwent Mckewon esophagectomy.

## 3 Surgical technique

Operation was started with right posterior lateral thoracotomy through the 5th or 6th intercostal space with division of lat dorsi and serratus anterior muscle. The deflated lung is retracted anteriorly for exposure of the posterior mediastinum. The pleura incised and azygous vein divided the esophagus is dissected circumferentially from the level of hiatus into the thoracic inlet. Paraesophageal and subcranial lymph node are incorporated with the specimen to release carefully the gastric tube from adhesions with the right lung and thoracic wall, the esophagus is further dissected bluntly, a chest tube is placed and thoracotomy is closed in layers.

For abdominal phase a supraumbilical incision is made division of falciform and left triangular ligament is made to retract the left lobe of liver, the right gastric artery is preserved. The abdominal esophagus and nodes dissected, the hiatus opened dissection performed the course of right gastroepiploic artery is determined, the greater curvature of stomach is then mobilized towards the pylorus the left gastric vascular pedicle divided adequate mobilization of stomach done, cervical phase a 6 cm incision was made along the anterior border of left sternocleidomastoid muscle starting at the sternal notch and extending to the cricoid cartilage the platysma and omohyoid muscle divided middle thyroid vein and inferior thyroid vein divide esophagus is further dissected into the superior mediastinum with gentle dissection the esophagus is divide with linear stapler in the neck incision preserving the cervical esophagus.

### 3.1 Conduit preparation

The stomach and thoracic esophagus delivered out of abdominal incision, lymphatic tissue and right gastric vessel are preserved; starting from the lesser curvature of the stomach the linear cutter stapler is fired towards the fundus of the stomach thus creating a 4–5 cm wide gastric conduit ensuring 5 cm distal to the tumor, the gastric conduit stapler line is then oversewn with a running 3-0 PDS, and the gastric conduit is gently delivered through the mediastinum into the neck.

### 3.2 Cervical anastomosis

**Fig. 1:**
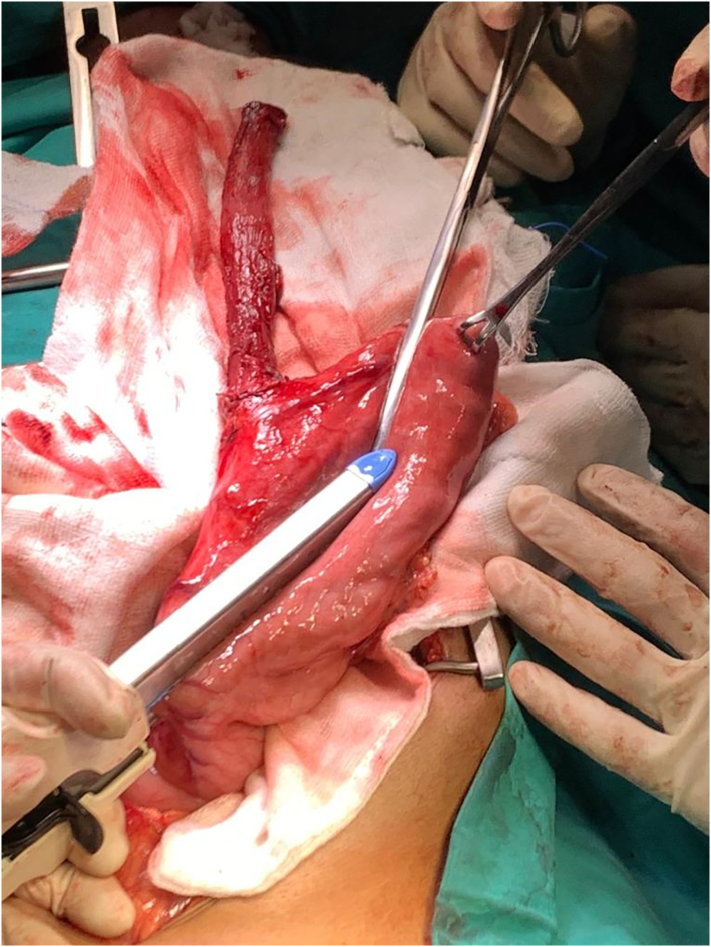
Linear cutter stapler gun is used to cut the lesser curvature of esophagus.

**Fig. 2:**
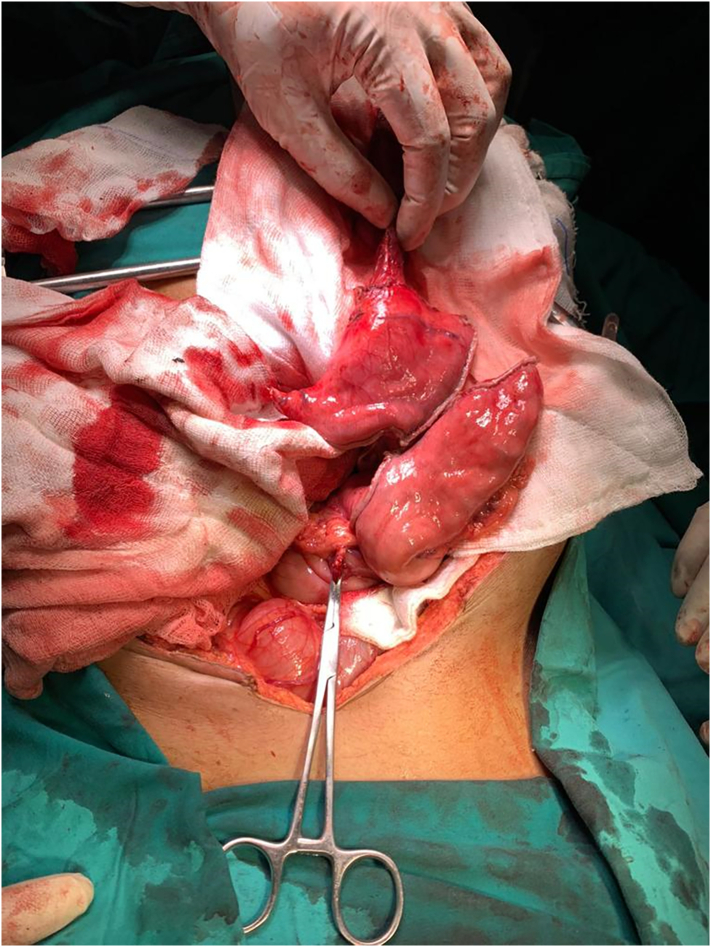
Esophagus and lesser curvature of stomach separated from rest of stomach.

**Fig. 3:**
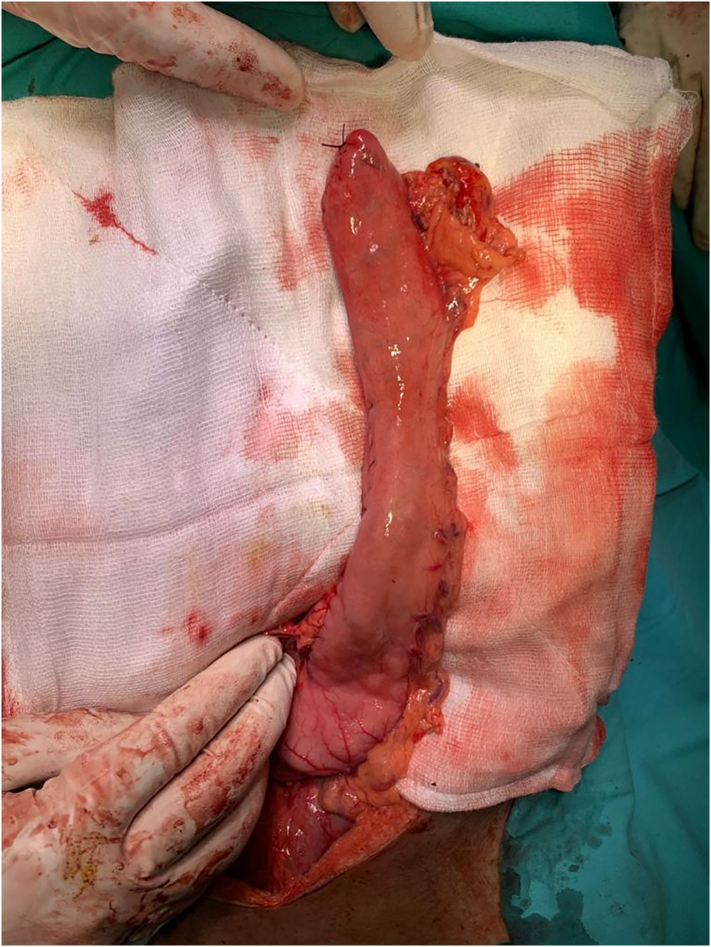
Conduit preparation starting from lesser curvature of stomach liner cutter stapler are fired towards the fundus of stomach thus creating a 4–5 cm wide gastric conduit ensuring 5 cm distal to the tumor.

**Fig. 4:**
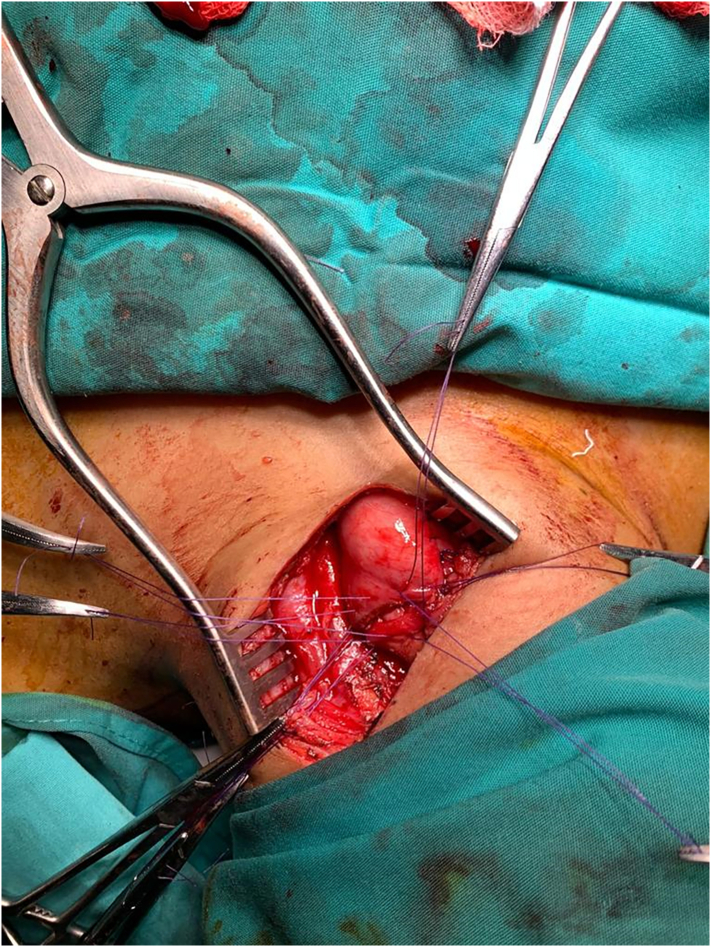
Cervical esophagus anastomosis.

**Fig. 5:**
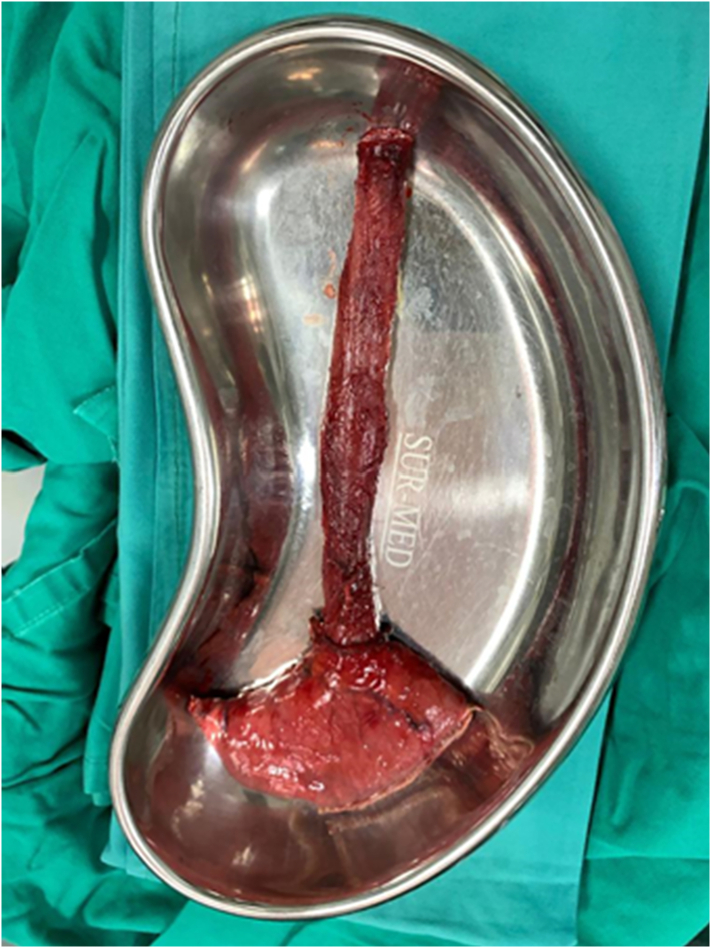
Removed gross specimen consists of esophagus and fundus of stomach.

A 45 mm long linear cutting stapler is placed in to cervical esophagus and gastric conduit to create posterior wall of anastomosis, an NG tube is placed through the anastomosis under direct visualization. The anterior aspect of anastomosis is completed with 4-0 PDS suture, a penrose drain is placed, the platysma is loosely approximated to sternocleidomastoid muscle with interrupted vicryl suture, the skin is closed. Feeding jejunostomy is located 20 com distal to duodeno jejunal junction (Fig. 1, Fig. 2, Fig. 3, Fig. 4, Fig. 5).

Gross pathology showed the tumor to be friable and having multiple ulcerations on the surface. H&E sections revealed a small, blue, round tumor. Histopathological examination showed positive CD99, CK (pan), Ki67 (70 %+), Fil-1, and CD34 levels.

## 4 Follow-up and outcome

The patient has been followed up regularly for every 3 month after surgery and for 6 month after adjuvant chemotherapy including clinical examination, ECT, chest CT, and gastroscopy. No obvious signs of recurrence or metastasis were found, and the patient's general condition was satisfactory.

## 5 Conclusion

In conclusion, we present an uncommon case of extraskeletal Ewing's sarcoma, and discuss its rare presentation and evolution. To our knowledge, this is the first reported case of paraesophageal primary Ewing's sarcoma.

## Consent

Written informed consent was obtained from the patient for publication of this case report.

## Provenance and peer review

Not commissioned, externally peer-reviewed.

## Ethical approval

It's a case report, no ethical approval required for this publication.

## Funding

N/A.

## Guarantor

Hina Khalid.

## Research registration number

None.

## CRediT authorship contribution statement

**Hina Khalid:** For manuscript writing, literature review, interpretation, data collection involve in surgery
**Niaz Hussain:** Review and analysis
**Rafay Shamshad:** Review and analysis.

## Declaration of competing interest

There are no conflicts of interest.
